# Evaluation of Fractional Exhaled Nitric Oxide in Pediatric Asthma and Allergic Rhinitis

**DOI:** 10.3390/children8010003

**Published:** 2020-12-23

**Authors:** Yoon Young Jang, Ji Young Ahn

**Affiliations:** 1 Department of Pediatrics, School of Medicine, Daegu Catholic University Medical Center, Daegu 42472, Korea; yyjang0117@gmail.com; 2 Department of Pediatrics, College of Medicine, Yeungnam University, Daegu 38541, Korea

**Keywords:** fractional exhaled nitric oxide, pediatric asthma, cutoff value

## Abstract

Fractional exhaled nitric oxide (FeNO) is a non-invasive test for evaluating the degree of airway inflammation and for the diagnosis, evaluation, and treatment of asthma. We attempted to measure FeNO levels in Korean children with asthma and determine its cutoff value for diagnosing asthma. We enrolled 176 children and adolescents between the ages of 5 and 18 years, who visited for the evaluation of chronic cough, shortness of breath, and wheezing. Among them, 138 patients who underwent skin prick tests or inhalation Immuno CAP (UniCAP; Pharmacia, Uppsala, Sweden) tests for allergy testing together with a pulmonary function test were included. FeNO was measured using a NIOX MINO (Aerocrine AB, Solna, Sweden) instrument according to the American Thoracic Society/European Respiratory Society (ATS/ERS) guidelines. There were 29 patients with asthma, 43 with rhinitis, and 38 with asthma and allergic rhinitis. In the asthma group, FeNO levels significantly correlated with total immunoglobulin E (r = 0.572, *p* < 0.001), but did not show significant correlation with pulmonary function test parameters (forced vital capacity—FVC, forced expiratory volume in one second—FEV1, FEV1/FVC) or PC20 (provocative concentration of methacholine causing a 20% fall in FEV1). The FeNO cutoff values obtained in the asthma and asthma rhinitis groups were 16.5 ppb and 18.5 ppb, respectively. Hence, we provide a FeNO cutoff value according to the presence or absence of rhinitis in pediatric patients with asthma.

## 1. Introduction

Asthma is a chronic inflammatory airway disease characterized by airway hyperresponsiveness and reversible airway obstruction. Pulmonary function tests have been widely used for the diagnosis and evaluation of the severity of asthma, as well as for guiding treatment, while sputum tests and bronchoalveolar lavage have been used to evaluate the degree of airway inflammation. In children, it is difficult to perform general sputum tests and bronchoalveolar lavage; therefore, some clinicians recommend the induced sputum test. However, young patients with poor cooperation often have difficulty undergoing these tests, and there are other drawbacks such as the need for equipment to conduct the test [1,2].

Fractional exhaled nitric oxide (FeNO) is a tool, that reflects the degree of eosinophilic airway inflammation and has the advantage of being relatively easy to perform and is non-invasive; it has therefore been increasingly utilized in recent years [3]. This test is especially useful for evaluating airway inflammation in children, in whom it is difficult to perform sputum collection or bronchoalveolar lavage [4]. However, since the measured value can be influenced by several factors, standard test guidelines were developed by the American Thoracic Society (ATS) and the European Respiratory Society (ERS) in 2005 [5] and 2011 [6], respectively. The cutoff values of FeNO published in these guidelines can be used for the diagnosis of asthma, to monitor asthma severity, and to establish treatment plans [7,8]. Since the interpretation of FeNO values varies according to race and age, continuous research is required to further our understanding of the clinical utility of FeNO in South Korean children and adolescents. Therefore, in this study, we attempted to measure FeNO levels in children with asthma in South Korea and determine its cutoff value for diagnosing asthma.

## 2. Materials and Methods

The study subjects included 176 children and adolescents between the ages of 5 and 18 years, who visited the Department of Pediatrics at two tertiary hospitals in Daegu for the evaluation of chronic cough, shortness of breath, and wheezing between January 2019 and December 2019. The medical records of the subjects were analyzed retrospectively. Participants were excluded if they did not undergo allergy testing with either a skin prick test or the inhalation Immuno CAP test, if they showed signs of acute infection, or if they had a history of previous asthma treatment. The final analysis included 138 patients. The patients were classified according to the results of allergy and methacholine challenge tests. We analyzed sex, height, weight, body mass index (BMI), serum total eosinophil count, serum total immunoglobulin E, specific IgE antibodies, skin prick test, pulmonary function test, methacholine challenge test, and FeNO levels in each group.

### 2.1. Blood Test

The serum total eosinophil count was measured using an automated hemocytometer, and serum total immunoglobulin E and specific IgE antibodies were measured using the CAP radioallergosorbent technique (UniCAP; Pharmacia, Uppsala, Sweden). For the specific IgE antibodies, six antigens were tested (Dermatophagoides farina (Der f), Dermatophagoides pteronyssinus (Der p), dog dander, cat dander, Alternaria, and Aspergillus fumigatus), and values of 0.35 kU/L or higher were defined as positive. Those who had positive results for any antigens on either specific IgE antibody or skin prick test were defined as the atopic group, while those who had negative results on the tests were defined as the non-atopic group.

### 2.2. Skin Prick Test

Skin prick tests were conducted on 34 types of antigens, including alder, ash, beech, birch, elm, hazel, oak, plane, willow, maple ash, poplar, chrysanthemum, Bermuda, timothy, olive, nettle, plantain, dandelion, rye, fat hen, ragweed, mugwort, Der f, Der p, guinea pig, horse, dog dander, cat dander, hamster, cockroach, Alternaria, Aspergillus, and Clostridium (Bencard Allergie GmbH, Munich, Germany). Histamine and normal saline were used as positive and negative controls, respectively, and the size of swelling was measured after 15 min. A positive result was defined as swelling of 3 mm or larger, or greater than that of the histamine control. None of the subjects received antihistamines in the 3 days prior to the test.

### 2.3. Pulmonary Function Tests

Forced vital capacity (FVC), forced expiratory volume in one second (FEV1), and FEV1/FVC were measured according to the ATS standard using a spirometer (Vmax 20; Viasys, San Diego, CA, USA). Measurements were taken three times, and the maximum values were used [9]. Percent predicted values were calculated based on the Third National Health and Nutrition Examination Survey [10]. None of the subjects received inhaled short-acting and long-acting β2-agonists in the 48 h prior to the test.

### 2.4. Methacholine Provocation Test

Provocholine (Methacholine Chloride USP; Apotex Pharmachem, Inc., Brantford, Ontario, Canada) was used, and FVC, FEV1, and PC20 (provocative concentration of methacholine causing a 20% fall in FEV1) were measured. For the methacholine provocation test, the 5-breath technique was used as in the ATS guidelines, and the concentrations were measured at 0.0625, 0.25, 1, 4, and 16 mg/mL [11]. Asthma was diagnosed when PC20 was less than 16 mg/mL in the methacholine provocation test.

### 2.5. Fractional Exhaled Nitric Oxide

FeNO was measured using a NIOX MINO (Aerocrine AB, Solna, Sweden) instrument, which uses an electrochemical measurement method. The test was conducted according to the ATS/ERS standard test guidelines [5], and the expiratory flow rate was maintained at 50 mL/s for more than 6 s in those aged 12 years or older, and for more than 4 s in those under 12 years of age. The tests were performed prior to the pulmonary function and methacholine provocation tests. Subjects avoided eating and drinking 2 h before the FeNO measurements.

### 2.6. Statistical Analysis

For statistical analysis, PASW Statistics ver. 18.0 (SPSS Inc., Chicago, IL, USA) was used. Quantitative values were expressed as means and standard deviations or as medians from minimum to maximum. Analysis of variance was conducted for comparison between groups for parametric variables, and the Kruskal–Wallis test was conducted for non-parametric variables. The Bonferroni test was used for post-hoc analysis. In the association analysis, Pearson correlation was used for parametric variables and Spearman correlation was used for non-parametric variables. Receiver-operating characteristic curve analysis was used to obtain the exhaled nitric oxide cutoff value corresponding to the highest sensitivity and specificity. A *p* value of less than 0.05 was considered statistically significant.

## 3. Results

Among the 138 patients, 90 (65.2%) were boys and 48 (34.8%) were girls with an average age of 10.4 years (10.4 ± 3.7). Sixty-seven patients had asthma and 81 patients had rhinitis, while 38 patients had both asthma and allergic rhinitis. In addition, 28 patients showed no specific findings in the pulmonary function and allergy tests.

The patients were classified according to the results of allergy and methacholine challenge tests, as follows: asthma only in the asthma group, rhinitis only in the rhinitis group, both asthma and rhinitis in the asthma rhinitis group, and neither asthma nor rhinitis in the control group. There was no statistically significant difference in age between the groups. The proportion of boys was significantly higher in the asthma rhinitis groups than in the control group (*p* < 0.001). There were no statistically significant differences in height, weight, and BMI between the groups, but there were statistically significant differences between the groups in total immunoglobulin E (*p* < 0.001) levels and the total number of positive skin prick tests (*p* < 0.001). There was a significant difference in total immunoglobulin E between the asthma rhinitis group and the control group (*p* = 0.002). There were significant differences in the total number of positive skin prick tests between the rhinitis and control groups (*p* = 0.001), and between the asthma rhinitis and control groups (*p* < 0.001). The highest median values of eosinophil count and total immunoglobulin E were found in the asthma rhinitis group. The highest mean value of the number of positive skin prick tests was found in the rhinitis group. With respect to pulmonary function test results, there was no statistically significant difference in FVC and FEV1 between the groups, while there was a statistically significant difference between groups in FEV1/FVC (*p* < 0.001) and PC20 (*p* = 0.004). There were significant differences in FEV1/FVC between the asthma and rhinitis groups (*p* = 0.009), between the asthma and the control groups (*p* = 0.030), between the asthma rhinitis and rhinitis groups (*p* < 0.001), and between the asthma rhinitis and control groups (*p* < 0.001). There were significant differences in PC20 between the asthma and rhinitis groups (*p* < 0.001), between the asthma and control groups (*p* < 0.001), between the asthma rhinitis and rhinitis groups (*p* < 0.001), and between the asthma rhinitis and control groups (*p* < 0.001). The lowest value of FEV1/FVC and PC20 was found in the asthma rhinitis group. The mean value of FeNO for all patients was 24.6 ppb. The highest median value of FeNO was found in the asthma rhinitis group, and there was a significant difference (*p* < 0.001) (Table 1). There were significant differences in FeNO between the asthma and control groups (*p* = 0.005), between the asthma rhinitis and rhinitis groups (*p* = 0.023), and between the asthma rhinitis and control groups (*p* < 0.001)

**Figure 1:**
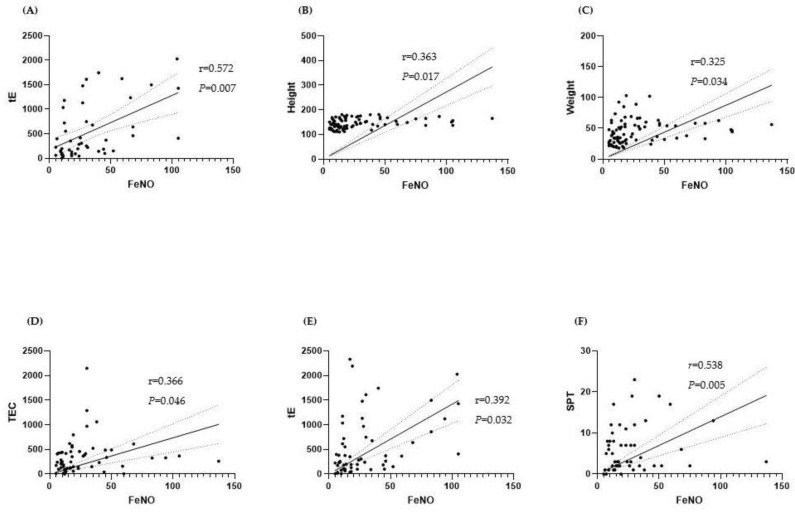
Correlations between FeNO and other factors in each group. (**A**) Correlations between FeNO and total immunoglobulin E in the asthma group. (**B**) Correlations between FeNO and height in the rhinitis group. (**C**) Correlations between FeNO and weight in the rhinitis group. (**D**) Correlation between FeNO and blood eosinophil count in the rhinitis group. (**E**) Correlation between FeNO and total immunoglobulin E in the rhinitis group. (**F**) Correlation between FeNO and the numbers of positive skin prick test in the rhinitis group. FeNO, fractional exhaled nitric oxide; BA, bronchial asthma; AR, allergic rhinitis; tE, total immunoglobulin E; TEC, total eosinophil count; SPT, skin prick test.

We checked correlations between FeNO and sex, age, height, weight, BMI, peripheral blood eosinophil count, total immunoglobulin E, number of positive skin prick tests, and number of positive Immuno CAP results in each group. In the asthma group, FeNO showed a statistically significant correlation with total immunoglobulin E (r = 0.572, *p* < 0.001); in the rhinitis group, it showed statistically significant correlation with height, weight, peripheral blood eosinophil counts, total immunoglobulin E, and the number of positive skin prick tests (Figure 1). In the control group, FeNO showed statistically significant correlation with age, height, and weight, but no significant correlation with the pulmonary function test results (FVC, FEV1, FEV1/FVC) or with PC20 in the asthma group.

**Figure 2:**
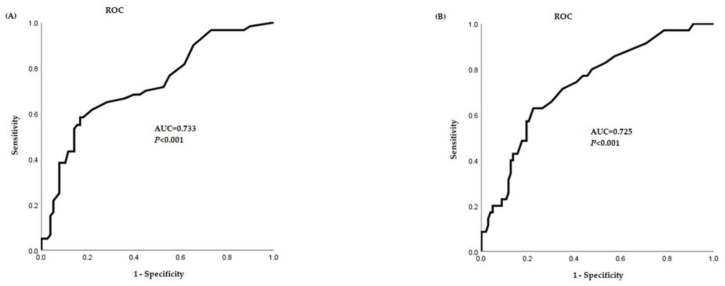
Receiver-operating characteristic (ROC) curve of FeNO cutoff value. (**A**) ROC curve of FeNO cutoff value in the asthma group. (**B**) ROC curve of FeNO cutoff value in the asthma rhinitis group. FeNO, fractional exhaled nitric oxide; AUC, area under curve.

The FeNO cutoff value obtained in the asthma group was 16.5 ppb (sensitivity 66.7%, specificity 64.1%, positive predictive value 62.9%, negative predictive value 60%), and the FeNO cutoff value in the asthma rhinitis group was 18.5 ppb (sensitivity 65.7%, specificity 69.9%, positive predictive value 62.7%, negative predictive value 70.3%) (Figure 2).

BA, bronchial asthma; AR, allergic rhinitis; BMI, body mass index; ECP, eosinophil cationic protein; FVC, forced vital capacity; FEV1, forced expiratory volume in one second; PC20, provocative concentration of methacholine causing a 20% fall in FEV1; FeNO, fractional exhaled nitric oxide; IgE: immunoglobulin E.

## 4. Discussion

This study provides FeNO cutoff values for the diagnosis of asthma according to the presence or absence of rhinitis in pediatric patients with asthma.

Exhaled nitric oxide is a biomarker of eosinophilic airway inflammation, and is known to be elevated in asthmatic patients [12]. Exhaled nitric oxide is produced by the bronchial epithelial cells through nitric oxide synthase 2, and increased inducible NO synthase expression has been demonstrated in asthmatic patients [12]. Previous studies have demonstrated a significant relationship between FeNO and eosinophilic inflammation in the sputum, airway mucosal biopsy, bronchoalveolar lavage fluid, and the eosinophil activation index, and have proposed that FeNO is an indicator of responsiveness to steroid treatment [13]. However, various factors such as airway infection, nitrate-containing food intake, smoking, exercise, steroids, leukotriene antagonists, and bronchodilators may affect the measured values of FeNO; therefore, careful consideration must be made to accurately interpret the results [2].

In previous studies, the measured value of FeNO in healthy people was reported to be approximately 5–25 ppb [14], and this is affected by race, age, sex, and co-morbidity [1]. In healthy patients younger than 12 years of age, FeNO is known to correlate with age; the correlation increases by approximately 5% with an increase in age [6,15]. This is thought to be due to the increase in NO synthase activity that occurs with airway development and maturation [16]. In a domestic study on patients with atopic asthma, there was no difference in FeNO according to age and sex [17]. The subjects for that study were children and adolescent patients with atopic asthma, and what is assumed that no changes in FeNO were observed with age due to the effects of atopy. In our study, FeNO correlated with age in the control group, but not in the atopic group. A previous study reported that FeNO reflects airway hypersensitivity and airway reversibility, but is not related to pulmonary function [18]. Some studies have also reported that FeNO has no association with PC20 [19]. This is thought to be due to the pulmonary function test being an index that reflects the diameter of the airway rather than airway inflammation [20], while FeNO is an index that reflects airway inflammation.

In our study, FeNO correlated with age, height, and weight in the control group [21], while in patients with asthma, there was a significant correlation with total immunoglobulin E [22]. However, there was no significant correlation between FeNO and pulmonary function test or PC20 values. In patients with rhinitis, FeNO showed significant correlation with peripheral blood eosinophil counts, total immunoglobulin E, and the number of positive skin prick tests; in the patients with asthma rhinitis, the FeNO value was higher than that of other groups. This is consistent with the results of previous studies, which reported that airway hypersensitivity was increased in cases of rhinitis and asthma [23] and that there was a relative increase in FeNO with more types of sensitizing allergens [24]. Our study showed that FeNO is more affected by allergic factors than age, height, weight, and body mass index in patients with atopy.

For diagnosing asthma and eosinophilic inflammation, cutoff values were provided by the ATS/ERS standard guidelines of 2011, which stated that in children and adolescents complaining of respiratory symptoms, the possibility of asthma was high when values were 35 ppb or more [2,6]. However, it is known that the positive predictive rate for the diagnosis of asthma is approximately 70%; therefore, asthma cannot be completely excluded in patients with low FeNO [25].

In our study, the cutoff values of FeNO in the asthma and asthma rhinitis groups were 16.5 ppb and 18.5 ppb, respectively. These values showed a difference from the FeNO cutoff value of domestic research published by Woo et al. (22 ppb, sensitivity 56.9%, specificity 87.2%, positive predictive value 90.5%, negative predictive value 48.6%); this may be due to the fact that the definition of asthma in the latter study was PC20 8 mg/mL or less [26]. Based on the findings of our study and that of previous studies, the FeNO cutoff value in South Korean pediatric and adolescent asthma patients differs from the 35 ppb suggested by the ATS/ERS. This may be attributed to the fact that FeNO is correlated with age or is related to differences in race. An alternative explanation may be related to differences in PC20 cutoff values. In this study, asthma was diagnosed based on a PC20 value of 16 mg/mL or less with the methacholine provocation test. When treating children and adolescents with asthma in South Korea, most of the criteria for airway hypersensitivity, which is a characteristic of asthma, include a PC20 of less than 16 mg/mL; however, a cutoff of 8 mg/mL or 25 mg/mL is used in some cases. For the proper establishment and utilization of FeNO, it is necessary to apply consistent criteria for the definition of airway hypersensitivity, which is a diagnostic criterion for asthma. Therefore, a cutoff value of FeNO can only be established by applying consistent criteria.

Our study had certain limitations. First, this was a retrospective study that analyzed the results of tests that had already been performed. Second, this study could not count the number of eosinophils in the sputum, which can be used to identify the actual degree of airway inflammation; thus, we were unable to clarify the association of this parameter with FeNO. Third, the results of this small-scale study cannot be generalized to all South Korean children and adolescents. Lastly, since children were enrolled after presenting to the hospital with specific respiratory complaints, these results may not be generalizable to all general pediatric patients who may also be cared for in the community. These cutoff values need to be validated in another cohort with healthy controls, who have no symptoms of cough, shortness of breath, or wheezing.

## 5. Conclusions

This study provides a FeNO cutoff value according to the presence or absence of rhinitis in pediatric patients with asthma. In addition, a PC20 value of 16 mg/mL, which is commonly used in clinical practice, was used as a criterion for airway hypersensitivity to determine the FeNO cutoff value. Further prospective research is necessary on large-scale cohorts.

## Figures and Tables

**Table 1:** Subject characteristics.

Parameters	BA (*n* = 29)	AR (*n* = 43)	BA + AR (*n* = 38)	Control (*n* = 28)	*p*-Value
Age (years)	8.9 ± 3.3	10.7 ± 3.7	10.9 ± 4.0	10.9 ± 3.6	0.124
Sex (male)	22	27	30	11	0.005
Height (cm)	135.0 ± 21.3	145.2 ± 20.7	145.7 ± 20.3	146.2 ± 20.6	0.114
Weight (kg)	38.9 ± 23.4	43.2 ± 18.4	46.0 ± 21.1	43.6 ± 16.6	0.550
BMI (z-score)	0.94 (−2.0–3.0)	0.64 (−1.5−2.4)	0.93 (−1.5−2.2)	0.35 (−1.4−2.4)	0.414
Eosinophil (/㎕)	142.5 (8.0−1930.0)	243.5 (17.0−620.0)	399.0 (0−2148.0)	133.5 (0−566.0)	0.085
Total IgE (IU/mL)	143.0 (70.6−335.5)	215.0 (97.0−440.3)	410.0 (195−1132)	58 (24.6−85.4)	<0.001
ECP (µg/L)	32.5 (12.5−62.8)	10.3 (3.8−16.2)	14.7 (8.3−23.1)	11.4 (3.0−50.3)	0.290
Number of positive skin prick test	2.4 ± 2.5 (*n*: 14)	6.1 ± 6.3 (*n*: 26)	5.6 ± 5.1 (*n*: 27)	0.7 ± 1.5 (*n*: 20)	<0.001
Number of positive CAP	1.5 ± 1.2 (*n*: 16)	1.9 ± 1.2 (*n*: 19)	1.9 ± 1.1 (*n*: 13)	1.1 ± 1.5 (*n*: 8)	0.385
FVC (%)	95.8 ± 16.0	95.0 ± 11.9	100.8 ± 17.5	98.9 ± 10.1	0.262
FEV1 (%)	94.0 ± 17.2	99.4 ± 13.8	94.0 ± 20.3	102.3 ± 12.8	0.116
FEV1/FVC	84.7 ± 9.1	91.1 ± 5.4	81.8 ± 9.5	91.1 ± 7.3	<0.001
PC20 (mg/mL)	6.4 (1.6−12.3)	25.0	5.7 (1.2−11.3)	23.7	0.004
FeNO(ppb)	19 (10−38)	13 (8−19)	27 (13−47)	11 (8−17)	<0.001

Values are expressed as means and standard deviations, or as medians from minimum to maximum. BA, bronchial asthma; AR, allergic rhinitis; BMI, body mass index; ECP, eosinophil cationic protein; FVC, forced vital capacity; FEV1, forced expiratory volume in one second; PC20, provocative concentration of methacholine causing a 20% fall in FEV1; FeNO, fractional exhaled nitric oxide; IgE: immunoglobulin E.

## Data Availability

The data presented in this study are available on request from the corresponding author. The data are not publicly available due to ethics.

## References

[1] Hahn Y.S. (2013). Measurements of fractional exhaled nitric oxide in pediatric asthma. Korean J. Pediatr..

[2] Kwon J.W., Song W.J., Kim M.H., Lim K.H., Yang M.S., Jung J.W., Lee J., Suh D.I., Shin Y.S., Kim S.-H. (2017). The KAAACI Standardization Committee Report on the procedure and application of fractional exhaled nitric oxide measurement. Allergy Asthma Respir. Dis..

[3] Berlyne G.S., Parameswaran K., Kamada D., Efthimiadis A., Hargreave F.E. (2000). A comparison of exhaled nitric oxide and induced sputum as markers of airway inflammation. J. Allergy Clin. Immunol..

[4] Warke T., Fitch P., Brown V., Taylor R., Lyons J., Ennis M., Shields M.D. (2002). Exhaled nitric oxide correlates with airway eosinophils in childhood asthma. Thorax.

[5] Society A.T. (2005). European Respiratory Society. ATS/ERS recommendations for standardized procedures for the online and offline measurement of exhaled lower respiratory nitric oxide and nasal nitric oxide, 2005. Am. J. Respir. Crit. Care Med..

[6] Dweik R.A., Boggs P.B., Erzurum S.C., Irvin C.G., Leigh M.W., Lundberg J.O., Olin A.-C., Plummer A.L., Taylor D.R., American Thoracic Society Committee on Interpretation of Exhaled Nitric Oxide Levels (FENO) for Clinical Applications (2011). An official ATS clinical practice guideline: Interpretation of exhaled nitric oxide levels (FENO) for clinical applications. Am. J. Respir. Crit. Care Med..

[7] Kang M.G., Yoon S., Sim J.H., Woo S.I. (2020). Fractional exhaled nitric oxide and forced expiratory volume in 1 second/forced vital capacity have predictive value of asthma exacerbation in Korean school children. Asia Pac. Allergy.

[8] White J., Paton J.Y., Niven R., Pinnock H. (2018). Guidelines for the diagnosis and management of asthma: A look at the key differences between BTS/SIGN and NICE. Thorax.

[9] Miller M.R., Hankinson J., Brusasco V., Burgos F., Casaburi R., Coates A., Crapo R., Enright P., van der Grinten C.P.M., Jensen R. (2005). Standardisation of spirometry. Eur. Respir. J..

[10] Hankinson J.L., Odencrantz J.R., Fedan K.B. (1999). Spirometric reference values from a sample of the general US population. Am. J. Respir. Crit. Care Med..

[11] Crapo R. (2000). Guidelines for methacholine and exercise challenge testing-1999. This official statement of the American Thoracic Society was adopted by the ATS Board of Directors, July 1999. Am. J. Respir. Crit. Care Med..

[12] Guo F.H., Comhair S.A., Zheng S., Dweik R.A., Eissa N.T., Thomassen M.J., Calhoun W., Erzurum S.C. (2000). Molecular mechanisms of increased nitric oxide (NO) in asthma: Evidence for transcriptional and post-translational regulation of NO synthesis. J. Immunol..

[13] Payne D.N., Adcock I.M., Wilson N.M., Oates T., Scallan M., Bush A. (2001). Relationship between exhaled nitric oxide and mucosal eosinophilic inflammation in children with difficult asthma, after treatment with oral prednisolone. Am. J. Respir. Crit. Care Med..

[14] Song W.J., Kwon J.W., Kim E.J., Lee S.M., Kim S.H., Lee S.Y., Kim S.H., Park H.W., Chang Y.S., Kim W.K. (2015). Clinical application of exhaled nitric oxide measurements in a Korean population. Allergy Asthma Immunol. Res..

[15] Kovesi T., Kulka R., Dales R. (2008). Exhaled nitric oxide concentration is affected by age, height, and race in healthy 9-to 12-year-old children. Chest.

[16] Buchvald F., Baraldi E., Carraro S., Gaston B., De Jongste J., Pijnenburg M.W., Silkoff P.E., Bisgaard H. (2005). Measurements of exhaled nitric oxide in healthy subjects age 4 to 17 years. J. Allergy Clin. Immunol..

[17] Kim J.O., Woo S.I., Hahn Y.S. (2011). Relevance of exhaled nitric oxide levels to asthma control test scores and spirometry values in children with atopic asthma. Pediatr. Allergy Respir. Dis..

[18] Steerenberg P., Janssen N., De Meer G., Fischer P., Nierkens S., Van Loveren H., Opperhuizen A., Brunekreef B., van Amsterdam J.G.C. (2003). Relationship between exhaled NO, respiratory symptoms, lung function, bronchial hyperresponsiveness, and blood eosinophilia in school children. Thorax.

[19] del Giudice M.M., Brunese F., Piacentini G., Pedullà M., Capristo C., Decimo F., Capristo A.F. (2004). Fractional exhaled nitric oxide (FENO), lung function and airway hyperresponsiveness in naïve atopic asthmatic children. J. Asthma.

[20] Covar R.A., Szefler S.J., Martin R.J., Sundstrom D., Silkoff P.E., Murphy J., Young D.A., Spahn J.D. (2003). Relations between exhaled nitric oxide and measures of disease activity among children with mild-to-moderate asthma. J. Pediatr..

[21] Horvath I., de Jongste J. (2010). Exhaled Biomarkers: European Respiratory Monograph.

[22] Janson C., Kalm-Stephens P., Foucard T., Norbäck D., Alving K., Nordvall S.L. (2005). Exhaled nitric oxide levels in school children in relation to IgE sensitisation and window pane condensation. Respir. Med..

[23] Ciprandi G., Tosca M.A., Capasso M. (2010). Exhaled nitric oxide in children with allergic rhinitis and/or asthma: A relationship with bronchial hyperreactivity. J. Asthma.

[24] Van Amsterdam J., Janssen N., De Meer G., Fischer P., Nierkens S., Van Loveren H., Opperhuizen A., Steerenberg P.A., Brunekreef B. (2003). The relationship between exhaled nitric oxide and allergic sensitization in a random sample of school children. Clin. Exp. Allergy.

[25] Karrasch S., Linde K., Rücker G., Sommer H., Karsch-Völk M., Kleijnen J., Jörres R.A., Schneider A. (2017). Accuracy of FENO for diagnosing asthma: A systematic review. Thorax.

[26] Woo S.I., Lee J.H., Kim H., Kang J.W., Sun Y.H., Hahn Y.S. (2012). Utility of fractional exhaled nitric oxide (FENO) measurements in diagnosing asthma. Respir. Med..

